# First Case of Tricuspid Valve Endocarditis Caused by *Gemella bergeri*

**DOI:** 10.1155/2015/704785

**Published:** 2015-07-30

**Authors:** Orathai Pachirat, George Watt, Burabha Pussadhamma

**Affiliations:** ^1^Division of Cardiovascular Medicine, Faculty of Medicine, Khon Kaen University, Khon Kaen 40002, Thailand; ^2^International Emerging Infection Program (IEIP), Thailand MOPH-US CDC Collaboration, Nonthaburi 10110, Thailand

## Abstract

*Gemella bergeri* is a Gram-positive cocci species arranged in pairs and composes the normal flora of oral cavity, digestive and urinary tract. Several species of *Gemella* are known to cause endocarditis. Here, we report the first case in Thailand of *G. bergeri* endocarditis whose blood culture was negative using routine methods but was positive by PCR identification of bacteria in the affected valve. A 37-year-old male presented with prolonged fever, weight loss, and dyspnea on exertion. By transthoracic echocardiography, he was suspected of having infective endocarditis of the tricuspid valve. The patient underwent tricuspid valve repair and vegetectomy. Routine hospital blood cultures were negative but *G. bergeri* was identified by PCR/sequencing of the heart valve tissue.

## 1. Introduction

*Gemella bergeri*, one of the six species belonging to the genus* Gemella*, is a family of Gram-positive cocci arranged in pairs and composes the normal flora of the oral cavity and digestive and urinary tract. It was isolated for the first time by Collins et al. in 1998 from the blood cultures of six febrile patients, three of whom had endocarditis [1]. Since then, only seven cases of* G. bergeri *endocarditis have been reported so far [1–4]. Predisposing cardiac abnormalities such as mitral valve prolapse, Tetralogy of Fallot, bicuspid aortic valve (BAV), and rheumatic heart disease seem to be the most important risk factors [2–5]. We describe herein a case of* G. bergeri* endocarditis of tricuspid valve with underlying tricuspid valve cleft. The causative pathogen was detected and identified by PCR/sequencing of the DNA of the cardiac valve tissue of the patient [6, 7].

## 2. Case Presentation

### 2.1. Patient Information

A 37-year-old male poultry farmer was admitted to a provincial hospital with prolonged fever for a month, weight loss, and shortness of breath and suspected of having infective endocarditis (IE). He was transferred to our cardiac center for evaluation. He denied any history of intravenous drug use, smoking, drinking alcohol, or cardiac abnormality.

### 2.2. Clinical Findings

On examination, his temperature was 38°C, blood pressure was 120/80 mmHg, respiratory rate was 20/min, and heart rate was 92 beats/min with a regular rhythm, no cyanosis with distension of the jugular vein, and prominent V wave. Cardiac examination revealed pansystolic murmur of grade 3/6 at left lower sternal area, mild hepatomegaly consistent with tricuspid regurgitation, and no signs of cardiac failure. Other systemic examinations showed no evidence of peripheral stigmata of IE.

### 2.3. Diagnostic Focus

**Figure 1:**
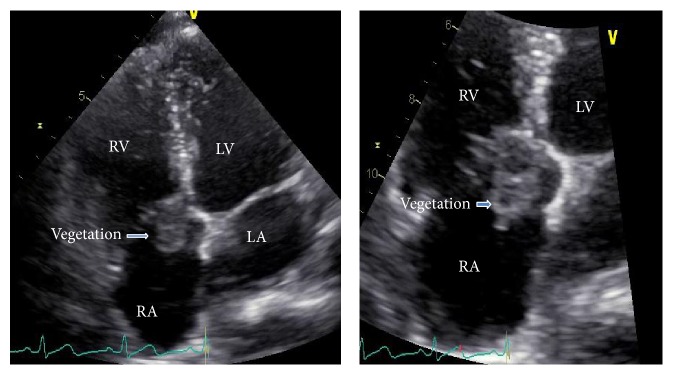
Transthoracic echocardiogram: four chambers' view of the heart showing large vegetation attached at tricuspid valve along right atrial surface. RA: right atrium; LA: left atrium; RV: right ventricle; LV: left ventricle.

Laboratory data included normal white blood cell count and blood chemistry. A transthoracic echocardiogram (TTE) revealed large mobile vegetation on the tricuspid valve (Figure 1) with moderately severe tricuspid regurgitation by Doppler-color flow and normal left ventricular function. Those findings were consistent with IE of the tricuspid valve. Three sets of routine aerobic blood cultures were negative.

### 2.4. Therapeutic Intervention and Follow-Up

The patient received intravenous antibiotics on the first day of hospitalization and was referred to cardiothoracic surgeons for surgical intervention due to unresolved fever after 8 days of medical treatment with large highly mobile vegetation; the patient underwent tricuspid valve repair with vegetectomy and it was found that the patient had tricuspid valve cleft as underlying heart disease.* G. bergeri* was demonstrated in heart valve tissue by real-time PCR. One month after surgery, the patient was discharged from the hospital with good outcome.

## 3. Discussion

*Gemella bergeri* is facultatively anaerobic, slow growing fastidious bacteria [8]. The importance of this organism and other* Gemella *species as a causative agent for human infectious diseases has been increasingly recognized.

One of the most recognized infections due to* Gemella *species is IE [9]. There are several cases reported in the literature that have highlighted the importance of preexisting heart valves disease or poor dental hygiene in patients [10–12]. It is highly sensitive to penicillin G or ampicillin and has low-level resistance to aminoglycosides. The outcome is generally good with appropriate antibiotic therapy [13].

To the best of our knowledge, there are only seven previously reported cases of IE due to* G. bergeri*. Collins et al. [1] reported a series of three cases of IE due to* G. bergeri* in 1998, which led to the identification of this bacterium as new species. Elsayed and Zhang [2] described another case of* G. bergeri* IE in an adult patient with bicuspid aortic valve that was complicated by valve ring abscess. Logan et al. [3] reported the first case of pediatric IE caused by* G. bergeri *in a boy with Tetralogy of Fallot and pulmonary atresia. Virgilio and Chieco [5] reported the sixth case of* G. bergeri* IE in an adult man with bicuspid aortic valve, using molecular diagnosis with 16S rRNA gene sequence analysis. Hussain et al. [4] reported neurological complications of* G. bergeri* IE in a young patient with rheumatic heart disease that was complicated by embolic stroke and rupture of mycotic aneurysm with bad outcome. All but the last reported case [4] showed good response to medical treatment and had good clinical outcome.

We describe herein the first case of* G*.* bergeri *IE in Thailand. All other previously reported cases of* G*.* bergeri *endocarditis in the literature had underlying cardiac conditions [2–5] and were the IE of the left side of the heart. Our patient has tricuspid valve cleft with the first case of right sided endocarditis which highlights the importance of molecular methods for the identification of microorganism directly from the heart valve tissue especially in blood culture-negative IE cases.
